# Sulfur Dioxide Enhances Endogenous Hydrogen Sulfide Accumulation and Alleviates Oxidative Stress Induced by Aluminum Stress in Germinating Wheat Seeds

**DOI:** 10.1155/2015/612363

**Published:** 2015-05-11

**Authors:** Dong-Bo Zhu, Kang-Di Hu, Xi-Kai Guo, Yong Liu, Lan-Ying Hu, Yan-Hong Li, Song-Hua Wang, Hua Zhang

**Affiliations:** ^1^School of Biotechnology and Food Engineering, Hefei University of Technology, Hefei 230009, China; ^2^Life Science College, Anhui Science and Technology University, Bengbu 233100, China

## Abstract

Aluminum ions are especially toxic to plants in acidic soils. Here we present evidences that SO_2_ protects germinating wheat grains against aluminum stress. SO_2_ donor (NaHSO_3_/Na_2_SO_3_) pretreatment at 1.2 mM reduced the accumulation of superoxide anion, hydrogen peroxide, and malondialdehyde, enhanced the activities of guaiacol peroxidase, catalase, and ascorbate peroxidase, and decreased the activity of lipoxygenase in germinating wheat grains exposed to Al stress. We also observed higher accumulation of hydrogen sulfide (H_2_S) in SO_2_-pretreated grain, suggesting the tight relation between sulfite and sulfide. Wheat grains geminated in water for 36 h were pretreated with or without 1 mM SO_2_ donor for 12 h prior to exposure to Al stress for 48 h and the ameliorating effects of SO_2_ on wheat radicles were studied. SO_2_ donor pretreatment reduced the content of reactive oxygen species, protected membrane integrity, and reduced Al accumulation in wheat radicles. Gene expression analysis showed that SO_2_ donor pretreatment decreased the expression of Al-responsive genes TaWali1, TaWali2, TaWali3, TaWali5, TaWali6, and TaALMT1 in radicles exposed to Al stress. These results suggested that SO_2_ could increase endogenous H_2_S accumulation and the antioxidant capability and decrease endogenous Al content in wheat grains to alleviate Al stress.

## 1. Introduction

Aluminum ions (Al^3+^) together with silicon and iron are the three most abundant mineral elements in soil. Whereas silicon and iron are required for plant growth, Al is toxic. Many different mechanisms have been advanced to explain Al toxicity in plants [1, 2]. One of the primary causes of Al toxicity is oxidative stress due to accumulation of reactive oxygen species (ROS), such as the superoxide anion (O_2_^•−^) and hydrogen peroxide (H_2_O_2_), bringing about lipid peroxidation in plant cells [3–5]. Plants have developed several strategies to counteract oxidative stress caused by Al, such as activation of antioxidants, and exudation of organic acids as a mechanism for Al exclusion [6]. Recently, a range of signaling molecules, such as inositol 1,4,5-triphosphate (IP_3_), salicylic acid, hydrogen peroxide (H_2_O_2_) and nitric oxide (NO), carbon monoxide (CO), and hydrogen sulfide (H_2_S), were found to participate in plant's resistance to Al-induced oxidative stress [4, 7–10].

Sulfur dioxide (SO_2_) is a colorless, nonflammable gas with a penetrating odor. Low concentrations of SO_2_ have been found to play a physiological role* in vivo* in animal models, participating in various biological processes [11]. The physiological processes regulated by SO_2_ in animals include cardiac function [11], inhibition of L-calcium channels in cardiomyocytes [12], and improvement in pulmonary vascular structural remodeling [13]. In plants, the toxic effects of SO_2_ on growth and development have been extensively studied [14, 15]. Exposure to high concentrations of SO_2_ can cause visible foliar damage, a decline in photosynthesis, an inhibition of plant growth, and structural disorganization and cell death [16–19]. On the other hand, many reports show that low levels of atmospheric SO_2_ might be beneficial to plants [20]. SO_2_ can be metabolized and used as a sulfur source for plant growth, especially when the sulfur supply in soil is insufficient for normal growth [20]. Recently, low concentrations of SO_2_ were found to induce transcriptome reprogramming associated with oxidative signaling and biotic defence responses in plants, suggesting a physiological role of SO_2_ in plant [21].

In plants, sulfate is taken up from soil by high-affinity transporters. Sulfate is largely transported to shoots where it can be activated by ATP via ATP sulfurylase in the leaves. The product is reduced by 5′-adenylylsulfate (APS) reductase to sulfite which can be reduced to H_2_S by sulfite reductase [22]. SO_2_ can also be produced endogenously from sulfur-containing amino acids [23]. The endogenous production of SO_2_ also suggests that it has a physiological role in plants.

In order to establish the role of SO_2_ in alleviating Al stress, we investigated the effects of SO_2_ pretreatment on H_2_S and ROS accumulation and the antioxidant system in whole wheat grains and in wheat radicles. We also analyzed endogenous H_2_S and Al content as a means of understanding the mechanism of the role of SO_2_. We speculated that SO_2_ might act as an antioxidant molecule to alleviate Al toxicity during wheat grain germination.

## 2. Materials and Methods

### 2.1. Materials and Treatments

Wheat (*Triticum aestivum* L.) grains were supplied by the Anhui Aidi Agricultural Technology Co., Ltd., Anhui Province, China. Sodium bisulfite (NaHSO_3_) and anhydrous sodium sulfite (Na_2_SO_3_) were used as sulfur dioxide (SO_2_) donors according to Laisk et al. [24]. Wheat grains were sterilized by 0.1% HgCl_2_ for 3 min and washed extensively with H_2_O and then dried with filter papers. Wheat grains of similar size were selected and allocated randomly in Petri dish (9 cm diameter × 1.2 cm depth, 50 grains per dish). Wheat grains were germinated in H_2_O or aqueous solutions of AlCl_3_ at 5, 10, 15, 20, 25, 30, 60, and 90 mM for 48 h at 25°C and the length of coleoptiles and radicles and radicle number were recorded. To test the protective role of SO_2_ on germination and seedling growth of wheat grains under Al stress, grains were pretreated with H_2_O or 0.4, 0.8, 1.2, 1.6, or 2.0 mM SO_2_ donor for 12 h and subsequently subjected to a semi-inhibitory AlCl_3_ concentration (15 mM). AlCl_3_ solutions were renewed every 12 h and geminating grains were sampled every 12 h for further analysis.

### 2.2. Determination of MDA, O_2_^•−^, and H_2_O_2_

The contents of MDA, O_2_^•−^, and H_2_O_2_ were determined by the method of Zhang et al. [25].

### 2.3. Assays of LOX, CAT, APX, and POD Activities

Activity of lipoxygenase (LOX, EC 1.13.11.12) was determined following the description by Surrey [26] and those of catalase (CAT, EC1.11.1.6), ascorbate peroxidase (APX, EC 1.11.1.11), and guaiacol peroxidase (POD, EC 1.11.1.7) were assayed according to Hu et al. [27]. Wheat grains were homogenized in ice-cold 50 mM phosphate buffer (pH 7.8) containing 1.0 mM EDTA. The homogenate was centrifuged at 15,000 g at 4°C for 10 min. The supernatant was used for activity determination.

### 2.4. Assays of Reducing Sugars and Soluble Protein

Wheat grains (0.5 ± 0.05 g) were ground in 5 mL of phosphate buffer (pH 7.0, 200 mM), the homogenate was centrifuged at 10,000 g for 30 min, and the supernatant was used for detection of reducing sugars and soluble protein content. Reducing sugar content was measured according to Miller [28].

For detection of soluble protein, 0.1 mL supernatant was mixed with 0.9 mL H_2_O and 5 mL Coomassie brilliant blue for 5 min and the absorbance recorded at 595 nm using the method described by Bradford [29].

### 2.5. Preparation of Wheat Radicles

Wheat grains were geminated in H_2_O for 36 h in the dark at 25°C and the average of radicle length was approximately 1.0 cm. The geminated wheat grains were pretreated with or without 1 mM SO_2_ donor for 12 h and then exposed to 0 or 400 *μ*M AlCl_3_ for 48 h.

### 2.6. Detection of Plasma Membrane Integrity, Al Accumulation, and ROS Production in Radicles

Plasma membrane integrity of wheat radicles was detected following the method of Yamamoto et al. [30]. Radicles were stained with Evans blue solution (0.025% [w/v] Evans blue in 100 *μ*M CaCl_2_, pH 5.6) for 10 min, then washed three times with 100 *μ*M CaCl_2_ solutions, and photographed. Staining intensity of Evans blue is positively correlated with more damaged plasma membrane.

Al content in radicles was visualized by staining tissues with hematoxylin. Hematoxylin stain was prepared as described by Polle et al. [31]. Wheat radicles were washed with H_2_O for 30 min and then stained with solution of 0.2% hematoxylin and 0.02% NaIO_3_ for 30 min at room temperature. Radicles were then immersed in H_2_O for 30 min to remove excess stain and photographed. Staining intensity of hematoxylin is positively correlated with Al uptake.

ROS distribution in radicle tips was detected by 2′,7′-dichlorofluorescin diacetate (DCFH-DA) following the method of LeBel et al. [32]. Radicle tips were incubated in a solution containing 100 *μ*M CaCl_2_ and 10 *μ*M DCFH-DA for 20 min and then washed three times with H_2_O. The fluorescence was detected with a Nikon 80i microscope (excitation at 488 nm and emission at 525 nm). For each treatment, ten individual roots from ten seedlings were examined and similar results were obtained.

### 2.7. Real-Time Quantitative RT-PCR Analysis in Wheat Radicles

Radicle tips were prepared for RNA extraction according to Li et al. [33]. Total RNA was isolated by grinding with liquid nitrogen according to the manufacturer's instructions (CWBIO, Beijing, China). cDNA was generated from total RNA with a reverse transcription kit (Prime Script RT Master Mix, Takara, Kyoto, Japan). Quantitative PCR was performed using a StepOnePlus Real-Time PCR System (Applied Biosystems, Foster City, CA, USA) with SYBR Premix Ex Taq (TaKaRa Bio Inc., China) according to the manufacturer's instructions. cDNA was amplified by PCR using the following primers: Ta*β*-actin forward (5′-CTATCCTTCGTTTGGACCTT-3′) and reserve (5′-AGCGAGCTTCTCCTTTATGT-3′); TaWali1 forward (5′-CTGATGGAGTCGAGCAAGG-3′) and reserve (5′-CCGAAGTAGCGATTTAGGAGT-3′); TaWali2 forward (5′-AGCCTACTGCTCCGCCTTGT-3′) and reserve (5′-CGTTTCGTCGGCATCTCC-3′); TaWali3 forward (5′-GACGAGCCCTAAGAAGACG-3′) and reserve (5′-CACGGAGCAATGACAACAG-3′); TaWali5 forward (5′-TGGACCCTGCAAGAAGTAC-3′) and reserve (5′-GCTGAACAACAAGCAACACC-3′); TaWali6 forward (5′-TACGGAATAGACAGGACAAGG-3′) and reserve (5′-CAGCATTTCGGGAACTCG-3′); TaALMT1 forward (5′-TGCCACGCTGAGTAAAGG-3′) and reserve (5′-CGCTGACGCTACGAAGAA-3′). Relative gene expression was presented as values relative to the corresponding gene expression in control, after normalization to the control Ta*β*-actin transcript levels.

### 2.8. Statistical Analysis

Statistical significance was tested by one-way ANOVA, and the results are expressed as the mean values ± SD (standard deviation) of three independent experiments. Each experiment was repeated three times.

## 3. Results

### 3.1. Inhibitory Effect of Al on Wheat Grain Germination

The effect of Al stress on wheat seedling growth and development was examined following incubation of grain in AlCl_3_ with concentrations ranging from 5 mM to 90 mM (Table 1). At concentrations of 5 mM or below, germination percentage, coleoptile length, and radicle number are almost unaffected, but radicle length was reduced by 13%, suggesting that the radicle is the primary target of Al toxicity. At 15 mM Al, germination percentage was almost halved compared with that of control and this concentration was selected for further experiments. At 90 mM Al, radicle growth was completely inhibited, but very stunted coleoptile growth was still observed.

### 3.2. SO_2_ Donor Ameliorates Al Stress in Germinating Wheat Grain

**Figure 1:**
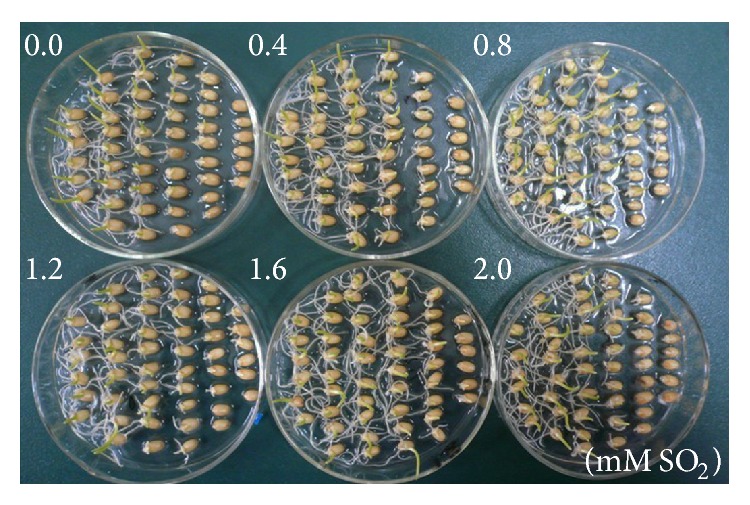
Effects of SO_2_ pretreatment on wheat grain germination under 15 mM Al stress. Wheat grains were pretreated with 0, 0.4, 0.8, 1.2, 1.6, and 2.0 mM SO_2_ for 12 h, subsequently subjected to 15 mM Al for further 48 h, and then photographed.

To establish whether the SO_2_ donor Na_2_SO_3_/NaHSO_3_ had a toxic effect on wheat grain germination, grains were germinated in different SO_2_ donor concentrations ranging from 0.4 to 2.0 mM for 36 h (see Table  S1 in Supplementary Material available online at http://dx.doi.org/10.1155/2015/612363). Table  S1 shows there was no significant change in germination percentage, coleoptile length, radicle length, or radicle number between water control and SO_2_ donor treatment, establishing that the concentrations of SO_2_ donor used in this work exhibited no visible toxic effects. To test the ability of SO_2_ donor to alleviate Al stress, wheat grains were pretreated with SO_2_ donor concentrations ranging from 0.4 to 2.0 mM for 12 h prior to incubation with 15 mM Al (Table 2 and Figure 1). At all SO_2_ donor concentrations used, SO_2_ pretreatment was effective in alleviating the toxic effects of Al in a dose dependent manner. The optimal SO_2_ donor concentration for alleviating Al stress was 1.2 mM, a concentration where the germination percentage was increased by 51%, radicle and coleoptile length by 28% and 26%, respectively, compared with those exposed to Al. This result clearly shows that SO_2_ alleviates Al-induced inhibition of wheat grain germination and seedling growth.

### 3.3. Effect of SO_2_ Donor on the Contents of Reducing Sugars and Soluble Protein in Al-Stressed Wheat Grain

**Figure 2:**
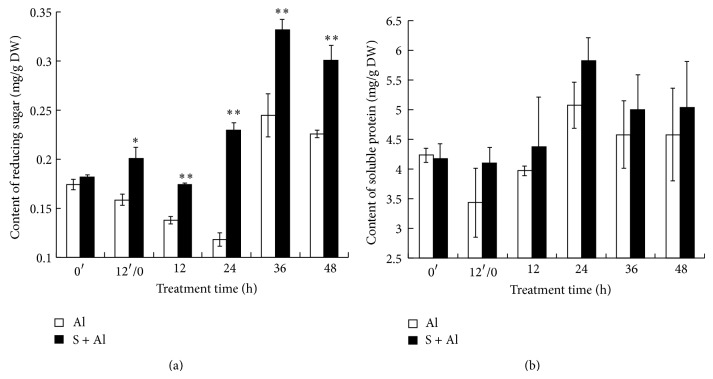
Effect of SO_2_ pretreatment on the contents of reducing sugar and soluble protein in Al-treated grain as shown in (a) and (b), respectively. Wheat grains were pretreated with water (Al) or 1.2 mM SO_2_ donor (S + Al) for 12 h (shown from 0′ to 12′/0 h of pretreatment time) and then exposed to 15 mM Al for further 48 h (shown as 12′/0, 12, 24, 36, and 48 h). The symbols ∗ and ∗∗ in this figure and following ones stand for significant difference between Al-treated grains with and without SO_2_ pretreatment at *P* < 0.05 and *P* < 0.01, respectively.

Figure 2(a) shows the changes in reducing sugars in germinating wheat grains preincubated in SO_2_ donor or H_2_O for 12 h followed by incubation in Al for 48 h. Within 12 h pretreatment in H_2_O and 24 h of Al treatment, the content of reducing sugar decreased gradually, whereas reducing sugar in the SO_2_ donor pretreatment remained stable and slightly increased at 24 h. Thereafter reducing sugar content increased steadily in both treatments followed by a slight decrease at 48 h. The content of reducing sugars in SO_2_ donor pretreated grain was always significantly higher than the counterpart of only Al treatment.

The content of soluble protein increased gradually and peaked on 24 h of Al stress followed by a slight decrease (Figure 2(b)). Though the mean values of soluble protein in SO_2_ donor pretreatment were higher than those pretreated in H_2_O, they are not significantly different.

### 3.4. Effect of SO_2_ Donor Pretreatment on Contents of Endogenous H_2_S, O_2_^•−^, H_2_O_2_, and MDA

**Figure 3:**
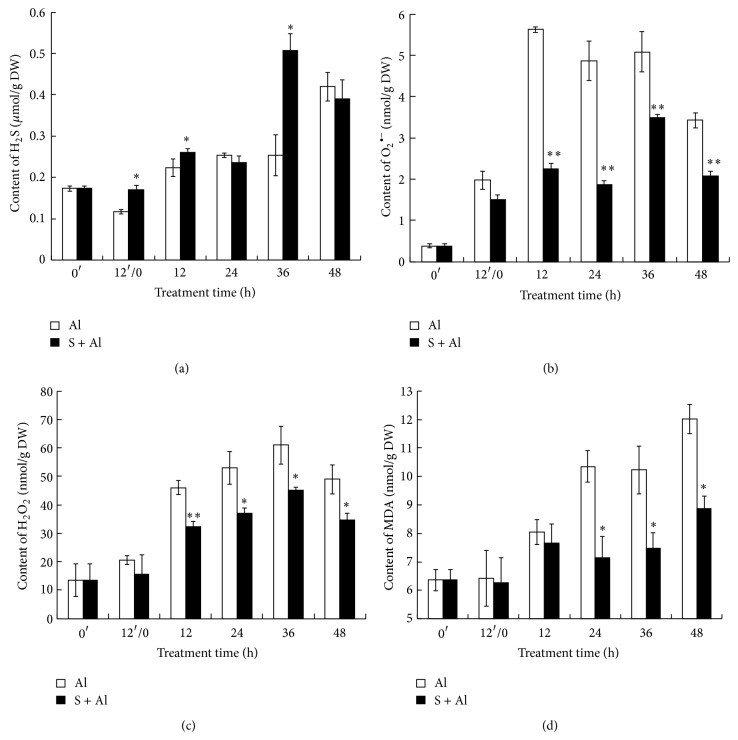
Effects of SO_2_ pretreatment on the accumulation of endogenous H_2_S (a), superoxide anion (O_2_^•−^) (b), hydrogen peroxide (H_2_O_2_) (c), and malondialdehyde (MDA) (d) in germinating wheat grains under Al stress. The numbers (0′, 12′/0, 12, 24, 36, and 48) or letters (CK or SO_2_) presented are the same as mentioned in Figure 2. Al: Al stress without SO_2_ pretreatment; S + Al: Al stress with SO_2_ pretreatment.

H_2_S, which can be produced from sulfite, is involved in plant growth regulation including various abiotic stresses [8, 22]. To investigate whether exogenous SO_2_ application can induce endogenous H_2_S production, we measured the concentration of H_2_S in Al-stressed wheat grain. Generally, H_2_S accumulated during wheat grain germination following pretreatment with water or SO_2_, but SO_2_ donor pretreatment significantly enhanced H_2_S concentration at 12 h of pretreatment and 12 h, 36 h of Al stress (Figure 3(a)).

To study the protective role of SO_2_ in the Al-stressed wheat grain, reactive oxygen species O_2_^•−^, H_2_O_2_, and malondialdehyde (MDA) were determined with time. As shown in Figure 3(b), a rapid accumulation of O_2_^•−^ was observed when H_2_O-pretreated grains were exposed to Al. During the first 12 h of Al exposure, the increase in O_2_^•−^ content was very rapid, but this was followed by a slow decrease. In contrast, the content of O_2_^•−^ in SO_2_ pretreatment increased slowly till 36 h of Al stress followed by a decrease. SO_2_ pretreatment maintained significantly lower level of O_2_^•−^ in Al-stressed wheat grains compared with grains incubated in H_2_O and exposed to Al.

H_2_O_2_ in both treatments increased gradually during pretreatment time and 36 h of Al stress and decreased at 48 h (Figure 3(c)). However, H_2_O_2_ content in SO_2_ pretreatment was significantly lower than that in water pretreatment when exposed to Al stress.

During the 12 h pretreatment time, no significant difference was observed in MDA content in wheat grains whether pretreated with SO_2_ donor or H_2_O (Figure 3(d)). After exposure to Al, the content of MDA in water pretreated grains increased rapidly till 48 h of Al stress. An increase of MDA content was also observed in SO_2_ pretreatment at 12 h of Al stress, but thereafter MDA content remained stable until 36 h. SO_2_ pretreatment dramatically reduced the amount of MDA from 24 h to 48 h of Al stress in comparison with grains pretreated in water.

### 3.5. Effects of SO_2_ Donor Pretreatment on POD, CAT, APX, and LOX Activities

**Figure 4:**
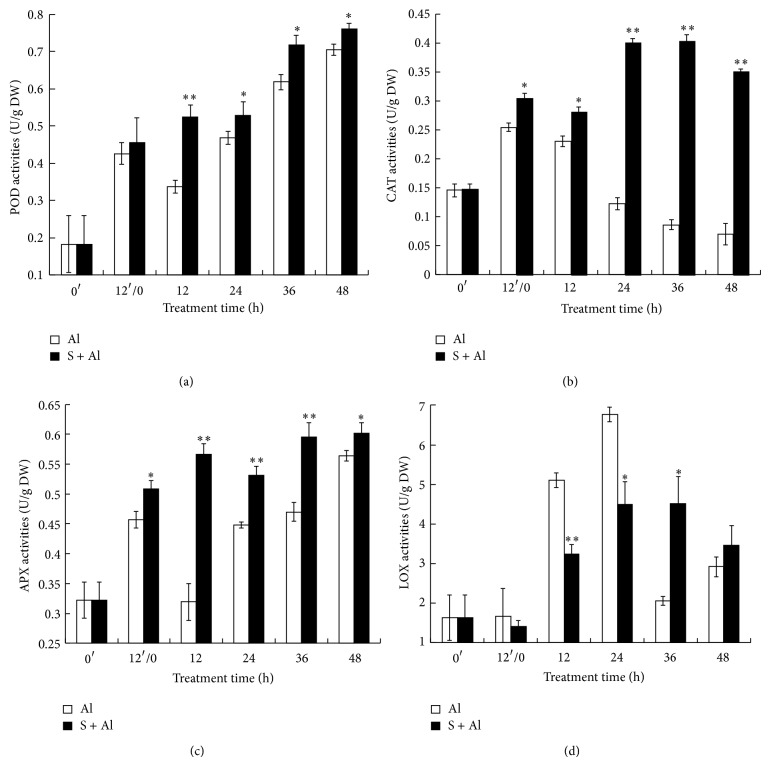
Effect of SO_2_ donor pretreatment on the activities of POD (a), CAT (b), APX (c), and LOX (d) in germinating wheat grains under 15 mM Al stress. Grains were treated and the number or letters presented are the same as mentioned in Figure 2. Al: Al stress without SO_2_ pretreatment; S + Al: Al stress with SO_2_ pretreatment.

Activities of POD, CAT, APX, and LOX were determined with time in SO_2_ donor and H_2_O-pretreated grains exposed to Al (Figure 4).  Figure 4(a) shows the time course of POD activity following pretreatment in SO_2_ donor or H_2_O for 12 h when POD activity showed almost a twofold increase. During Al stress, POD activity exhibited a gradual increase in both treatments, but SO_2_ pretreatment maintained significantly higher level of POD activity during Al stress.

The activity of CAT increased almost twofold during 12 h pretreatment with H_2_O or SO_2_ donor (Figure 4(b)). After exposure to Al, CAT activity in water pretreatment decreased gradually till 48 h of Al stress, suggesting that CAT activity is very sensitive to Al stress. In contrast, CAT activity in SO_2_ pretreatment increased steadily and decreased only slightly at 48 h of Al stress.

As shown in Figure 4(c), SO_2_ pretreatment enhanced APX activity in Al-stressed wheat grain. A rapid increase in APX activity occurred during the pretreatment time in H_2_O and SO_2_. Within the first 12 h of Al stress, APX activity in H_2_O-pretreated grains decreased sharply, whereas SO_2_ donor pretreatment enhanced APX activity slightly. Thereafter APX activity increased steadily in water pretreated grain, whereas its activity in SO_2_ donor pretreatment fluctuated slightly. The APX activity in SO_2_ donor pretreated grains was always significantly higher than the counterpart of water pretreatment.

An increase in LOX activity was observed during the first 24 h of Al stress in SO_2_ and H_2_O-pretreated grains (Figure 4(d)). However, the increase of LOX activity in water pretreatment was more rapid than after SO_2_ pretreatment. Thereafter LOX activity in water pretreatment showed a sharp decrease at 36 h of Al stress, while its activity in SO_2_ pretreatment decreased at 48 h. At 12 and 24 h of Al stress, SO_2_ pretreatment maintained significantly lower level of LOX, while at 36 h LOX activity in SO_2_ pretreatment was higher than that of water pretreatment.

### 3.6. Effects of SO_2_ Donor Pretreatment on Localization of Al, Lipid Peroxidation, and ROS Production

**Figure 5:**
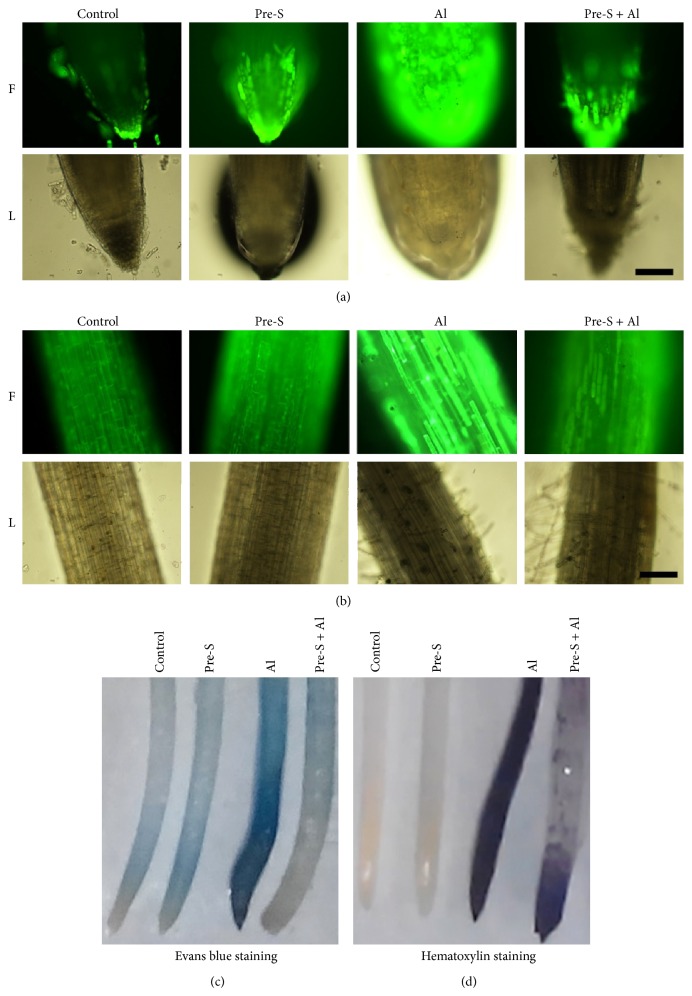
ROS staining ((a) on radicle tips; (b) on maturation zone; bar: 200 *μ*m), Evans blue staining (c), and hematoxylin staining (d) in wheat radicles. Initially, wheat grains were geminated in water for 36 h. Then four treatment groups were done as follows, control, 60 h in H_2_O; Pre-S, pretreatment with 1 mM SO_2_ donor for 12 h, and then exposed to H_2_O for 48 h; Al, 12 h in H_2_O prior to exposure to 400 *μ*M AlCl_3_ for 48 h; Pre-S + Al, 12 h in 1 mM SO_2_ donor pretreatment followed by 400 *μ*M AlCl_3_ for 48 h.

To detect ROS production in the radicle tips, we used DCFH-DA fluorescence to indicate ROS accumulation. As shown in Figure 5(a), Al treatment induced higher level of ROS in radicle as intense DCFH-DA fluorescence, while SO_2_ donor pretreatment for 12 h followed by Al stress significantly reduced fluorescence.  Figure 5(b) shows DCFH-DA fluorescence in maturation zone in radicles. Similarly, intense fluorescence in SO_2_ donor pretreatment followed by Al stress was much weaker than that in water pretreated plus Al-stressed radicles, suggesting that SO_2_ donor was effective in alleviating oxidative stress in radicles. SO_2_ donor treatment alone showed comparable fluorescence intensity as observed in water control.

The radicles were stained with Evans blue to show membrane integrity. The radicles treated with Al alone were stained extensively with Evans blue, while Al-stressed radicles pretreated with SO_2_ donor for 12 h were less stained (Figure 5(c)), suggesting SO_2_ donor serves to protect cell membrane from Al-induced damage. SO_2_ donor treatment alone showed similar Evans blue staining to water control, suggesting no visible damaging effect of SO_2_ on radicles.

The hematoxylin staining was used to detect Al accumulation in radicles. As shown in Figure 5(d), the radicles of water control and SO_2_ treatment incubated with hematoxylin showed no dark staining but wheat radicles treated with Al alone were stained intensively. In contrast, radicles pretreated with SO_2_ donor for 12 h and then exposed to Al for 48 h showed much weaker staining compared with Al stress, especially in the elongation zone.

### 3.7. Effect of SO_2_ Donor Pretreatment on the Relative Expressions of Aluminum Stress Related Genes

**Figure 6:**
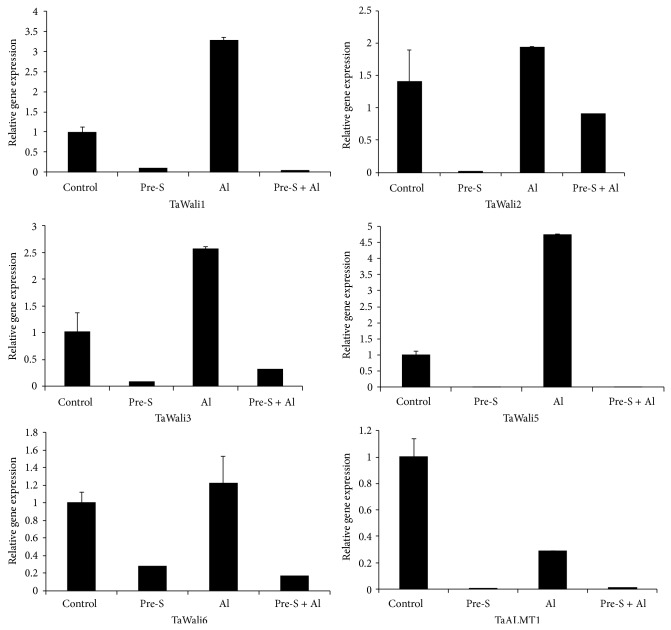
Effect of SO_2_ donor pretreatment on relative gene expression of TaWali1, TaWali2, TaWali3, TaWali5, TaWali6, and TaALMT1 in wheat radicals exposed to Al stress. Initially, wheat grains were geminated in water for 36 h. Then four treatment groups were done as follows, control, 60 h in H_2_O; Pre-S, pretreatment with 1 mM SO_2_ donor for 12 h, and then exposed to H_2_O for 48 h; Al, 12 h in H_2_O prior to exposure to 400 *μ*M AlCl_3_ for 48 h; Pre-S + Al, 12 h in 1 mM SO_2_ donor pretreatment followed by 400 *μ*M AlCl_3_ for 48 h.

We determined the changes in gene expression of aluminum stress related genes in wheat radicles. Radicles were pretreated with or without 1 mM SO_2_ donors for 12 h and then exposed to Al for 48 h. As shown in Figure 6, Al stress induced higher expression of TaWali1, TaWali2, TaWali3, TaWali5, and TaWali6 (wheat aluminum induced) in radicles, while pretreatment with SO_2_ donor for 12 h followed by Al stress alleviated such expression increase. Besides, the gene expression of TaALMT1 (Al-activated malate transporter) was also attenuated by SO_2_ pretreatment.

## 4. Discussion

In solution, SO_2_ is dissociated from its sulfite derivatives (NaHSO_3_/Na_2_SO_3_ 1 : 3 M/M) [34]. Thus NaHSO_3_/Na_2_SO_3_ (1 : 3 M/M) was chosen as an SO_2_ donor in our study. Similar to the observation that H_2_S could promote wheat grain germination and alleviate oxidative damage against Al stress [8], our results show that SO_2_ donor pretreatment alleviates Al stress in germinating wheat seedlings. Wheat grains pretreated for 12 h with the SO_2_ donor show an increase in germination percentage, coleoptile length, radicle length, and radicle numbers of wheat. The increase in the contents of reducing sugars and soluble protein suggests that nutrients in wheat grains pretreated with SO_2_ donor are rapidly mobilized to provide energy to grain germination. SO_2_ donor maintained lower level of H_2_O_2_, O_2_^•−^, and MDA probably by activation of the antioxidant system. These results suggest that SO_2_ acts as an antioxidant and may function in a way that is similar to what the effects of H_2_S, CO, and NO do in plants exposed to heavy metal stress [10, 35].

Sulfite can be reduced by sulfite reductase to H_2_S, which is incorporated into O-acetylserine via O-acetyl(thiol)lyase to form cysteine [22]. In RNA interfered mutant of sulfite reductase (SiR), sulfide synthesis in younger leaves was decreased by the impaired SiR activity [36]. In the present study, exogenous SO_2_ application can induce endogenous H_2_S production in Al-stressed wheat grains (Figure 3(a)), suggesting the interplay between sulfite and the formation of H_2_S.

Consistent with previous observations [7], our results show that Al stress caused overproduction of ROS in wheat. To mitigate and repair oxidative damage, plants have evolved an efficient antioxidant system that includes enzymes such as SOD, CAT, and APX that function to scavenge ROS [37]. SOD catalyzes the dismutation of the superoxide radical O_2_^•−^ and H^+^ into H_2_O_2_. CAT, APX, and POD are responsible for the elimination of H_2_O_2_ generated by SOD. Al stress brings about a dramatic increase in H_2_O_2_ and O_2_^•−^. The elevated levels of H_2_O_2_ and O_2_^•−^ suggest that antioxidant enzymes in Al-stressed wheat do not efficiently scavenge the overproduction of ROS, and this can result in lipid peroxidation or plasma membrane inhibiting grain germination and seedling growth [8]. Our data show that pretreatment of wheat with SO_2_ donor activates antioxidant enzymes including POD, CAT, and APX.

LOX, which catalyzes oxygenation of polyunsaturated fatty acids into lipid hydroperoxides, is considered an indicator of oxidative stress during responses to various environmental stresses [9]. Pretreatment with SO_2_ donor lowers LOX activity in Al-stressed wheat radicles compared to seedlings pretreated with H_2_O and exposed to Al. The lowering of LOX by SO_2_ pretreatment also helps to explain the lower MDA content of Al-stressed grain. Taken together, these data suggest that SO_2_ donor reduced oxidative stress by modulation of the antioxidant system.

Our data indicate that the radicle is the primary target for Al toxicity. DCFH-DA fluorescence assay shows that Al incubation induces higher accumulation of ROS in radicle tips and maturation zone. SO_2_ donor pretreatment effectively reduces ROS content in subsequent Al stress, suggesting the role of SO_2_ in alleviating oxidative stress. Correspondently, Al stress causes membrane injury to radicles, while SO_2_ donor effectively alleviates such injury. To understand whether SO_2_ donor helps to reduce Al accumulation in radicles, hematoxylin staining was used to indicate Al and the results show that SO_2_ donor obviously reduces Al content in radicles, implying a potential role of SO_2_ donor treatment as a strategy to reduce Al uptake.

In response to Al stress, many gene expressions are activated, for instance, TaWali (wheat aluminum induced), aluminum-activated malate transporter (TaALMT1) [38–41]. Relative gene expression analysis shows that Al treatment induces higher expression of TaWali, while these gene expression levels are reduced by SO_2_ donor pretreatment, suggesting the response to Al stress is attenuated in SO_2_ donor pretreatment.

## 5. Conclusion

In the present study, SO_2_ acts as an antioxidant signal to reduce ROS damage in wheat grains and radicles caused by Al stress. Besides, SO_2_ also decreases Al uptake. The induced higher level of H_2_S suggests an intricate interplay of SO_2_ and H_2_S in plants. Exogenous application of SO_2_ may be reduced to H_2_S by sulfite reductase, thus contributing to H_2_S production. H_2_S in itself acts as an antioxidant signaling molecule in plants' response to abiotic stress. Thus the nature of SO_2_/sulfite functions in alleviating Al stress still needs further research.

## Supplementary Material

Table S1 shows the germination percentages of wheat grains under SO2 donor treatment. Wheat grains were germinated in 0.0, 0.4, 0.8, 1.2, 1.6 or 2.0 mM SO_2_ donor for 36 h, and then the germination percentages are counted.

## Figures and Tables

**Table 1:** Inhibitory effect of Al stress on the germination of wheat grains. Wheat grains were exposed to 0, 5, 10, 15, 20, 25, 30, 60, or 90 mM AlCl_3_ for 48 h.

Al^3+^ concentration (mM)	Germination percentage (%)	Radicle length (cm)	Coleoptile length (cm)	Radicle number (50 grains)
0	64 ± 1.2^a^	3.1 ± 0.8^a^	1.5 ± 0.3^ab^	178 ± 7.8^a^
5	66 ± 1.1^a^	2.7 ± 0.5^ab^	1.6 ± 0.2^a^	168 ± 8.9^a^
10	51 ± 2.3^b^	1.9 ± 0.3^b^	1.3 ± 0.3^ab^	162 ± 7.6^a^
15	35 ± 3.8^c^	1.1 ± 0.2^c^	1.1 ± 0.2^bc^	142 ± 6.3^b^
20	28 ± 4.2^cd^	0.7 ± 0.3^cd^	0.8 ± 0.2^cd^	80 ± 5.6^c^
25	21 ± 5.1^de^	0.4 ± 0.3^d^	0.6 ± 0.2^de^	68 ± 6.1^c^
30	15 ± 4.7^ef^	0.2 ± 0.2^d^	0.3 ± 0.3^e^	45 ± 5.3^d^
60	8 ± 5.2^f^	0.1 ± 0.1^d^	0.3 ± 0.2^e^	22 ± 3.5^e^
90	7 ± 6.3^f^	0 ± 0^e^	0.2 ± 0.2^e^	0 ± 0^f^

Values are the means ± SD (*n* = 6). Values are the means ± SD (*n* = 6). Different letters mean significance of difference between different treatments (*P* < 0.05).

**Table 2:** Effects of SO_2_ donor pretreatment on wheat grain germination under 15 mM Al^3+^ stress. Wheat grains were pretreated with 0, 0.4, 0.8, 1.2, 1.6, and 2.0 mM SO_2 _ for 12 h and subsequently subjected to 15 mM AlCl_3_ for further 48 h, and then germination was investigated.

SO_2_ donor concentration (mM)	0.0	0.4	0.8	1.2	1.6	2.0
Germination percentage (%)	37 ± 3.3^a^	42 ± 2.7^a^	44 ± 3.5^a^	56 ± 3.8^a^	48 ± 2.7^a^	47 ± 3.1^a^
Length of radicle (cm)	1.42 ± 0.6^a^	1.72 ± 0.4^a^	1.80 ± 0.7^a^	1.82 ± 0.7^a^	1.78 ± 0.8^a^	1.62 ± 0.4^a^
Length of coleoptile (cm)	4.64 ± 0.4^a^	4.70 ± 0.6^a^	5.20 ± 0.5^a^	5.83 ± 0.8^a^	5.40 ± 0.8	5.23 ± 0.6^a^
Radicle number (50 grains)	127 ± 7.3^a^	135 ± 8.1^a^	139 ± 8.1^a^	148 ± 7.9^a^	130 ± 6.7^a^	119 ± 7.1^a^

Values are the means ± SD (*n* = 6). Different letters mean significance of difference between different treatments (*P* < 0.05).
