# Supplementary material for: Sulfur Dioxide Enhances Endogenous Hydrogen Sulfide Accumulation and Alleviates Oxidative Stress Induced by Aluminum Stress in Germinating Wheat Seeds
Source: Oxid Med Cell Longev. 2015 May 11;2015:612363. doi: 10.1155/2015/612363 (PMC4442301; doi:10.1155/2015/612363)
Supplement: Supplementary file 1 — Table S1 shows the germination percentages of wheat grains under SO2 donor treatment. Wheat grains were germinated in 0.0, 0.4, 0.8, 1.2, 1.6 or 2.0 mM SO2 donor for 36 h, and then the germination percentages are counted. [file 612363.f1.docx]

Table S1: Effects of SO_2_ donor treatment on wheat grain germination under normal condition. Wheat grains were cultured in 0.0, 0.4, 0.8, 1.2, 1.6, 2.0 mM SO_2_ donor for 36 h, and then the germination percentage are counted.

| SO_2_ donor concentration (mM) | 0.0 | 0.4 | 0.8 | 1．2 | 1.6 | 2.0 |
| --- | --- | --- | --- | --- | --- | --- |
| Germination percentage (%) | 64.4±2.1a | 69.2±3.2a | 67.7±3.4a | 71.3±5.6a | 73.4±5.3a | 70.2±4.7a |
| Length of radicle (cm) | 2.82±0.6a | 2.92±0.8a | 3.04±0.9a | 3.12±0.9a | 2.87±0.7a | 2.78±0.7a |
| Length of coleoptile (cm) | 4.04±0.3a | 4.20±0.3a | 4.60±0.4a | 5.03±0.7a | 4.70±0.4a | 4.43±0.3a |
| Radicle number (50 grain) | 97±8.7a | 105±8.9a | 109±7.9a | 115±10.6a | 110±8.6a | 99±7.5a |

Values are the means ± SD (n=6). Different letters mean significance of difference between different treatments (*P* ＜ 0.05).
